# Regenerative Endodontic Treatment in Dentinogenesis Imperfecta-Induced Apical Periodontitis

**DOI:** 10.1155/2024/5128588

**Published:** 2024-01-06

**Authors:** Ying Liao, Ting Pan, Xianghui Xing

**Affiliations:** Department of Pediatric Dentistry, Nanjing Stomatological Hospital, Medical School of Nanjing University, Nanjing, Jiangsu 210008, China

## Abstract

Pulp involvement of immature permanent teeth with dentinogenesis imperfecta is challenging and could lead to extraction. A case of dentinogenesis imperfecta-induced periapical periodontitis of an immature permanent tooth was treated with regenerative endodontic treatment (RET), and root maturation was observed in 12-month follow-up. An 8-year-old girl presented acute pain and swelling in central mandibular region. Clinical and radiographic examination revealed “shell teeth” appearance of teeth 31, 41, and 42. Periapical lesion of tooth 31 was observed. Tooth 41 was previously treated with apexification. RET was planned and carried out for the necrotic tooth (tooth 31) with dentinogenesis imperfecta. The 1-, 3-, 7-, and 12-month postoperative recall revealed complete healing of periapical lesions. Root maturation characterized by elongation of root, thickening of dentinal walls, and closure of root apex was observed with radiographic examinations. We show that RET could be a desirable treatment for necrotic immature permanent teeth with dentinogenesis imperfecta and lead to resolution of endodontic lesions as well as maturation of dental root. The findings of this case suggest that RET should be considered by endodontist and pediatric dentist to treat teeth with similar dental anomalies and apical periodontitis.

## 1. Introduction

Dentinogenesis imperfecta describes developmental defects of dentin caused by mutations in gene encoding dentin sialophosphoprotein (DSPP) [1]. Studies have shown that *Dspp* knockout mice exhibited severe dentin defects [2]. The dental phenotypes of the mutant mice could also be the result of the intracellular retention of mutant DSPP in the odontoblasts [3]. Epidemiological data showed that the incidence of dentinogenesis imperfecta ranges from 0.002% to 0.1% in different populations [4–6]. Both enamel and dentin development could be affected by dentinogenesis imperfecta. In mild cases, only crown discoloration and “thistle-shaped” pulp could be noticed. In more severe cases, attrition or even disappearance of crown and complete obliteration of pulp could be seen [7]. Enlarge pulp with “shell teeth” appearance could be scarcely found [8]. Pulp is rarely involved except for the most severe cases [9]. Due to the hypomineralized structure and poor prognosis, pulp involvement of dentinogenesis imperfecta-affected teeth could lead to extraction.

Regenerative endodontic treatment (RET) has been widely used in necrotic immature permanent teeth [10]. Compared to conventional root canal treatment, pulpotomy, and apexification, RET has the unique potential to continue root maturation and save the teeth for the lifetime of the patient [11]. The basis of RET relies on tissue engineering induced by a complex of stem cells and bioactive growth factors [12, 13]. Biomimetic scaffolds are also used in some cases to increase the success rate [14]. The success rate of RET has been reported to be 94.8% and 96% for dental trauma and caries-caused pulp necrosis, respectively [15]. The success rate of RET for teeth with developmental defects has only been assessed in teeth with dens evaginatus and was slightly lower (93%) [15]. RET for teeth with other dental anomalies, especially with dentinogenesis imperfecta, has been rarely reported.

This current case presents use of RET in a necrotic immature tooth with severe dentinogenesis imperfecta to induce root maturation and reduce risks of root fracture and extraction.

## 2. Case Presentation

**Figure 1:**
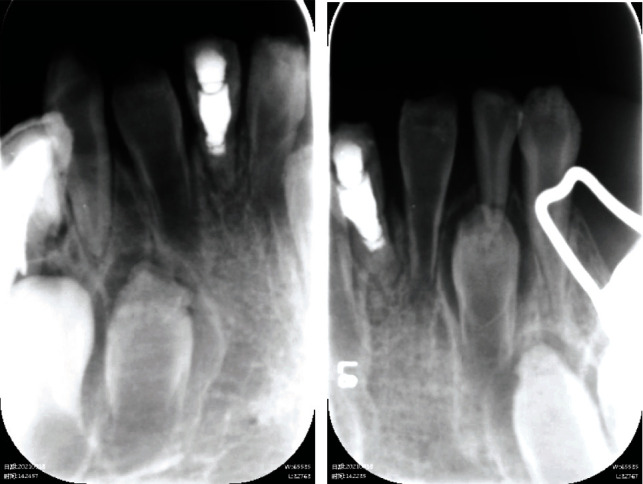
Preoperative radiographs. Short root, thin hard tissue, and enlarged pulp cavity was evident in teeth 31, 41, and 42. Pulp cavity of 41 was filled with radiopaque material. (a) Tooth 42 merely erupted, and periapical radiolucency was noticed. (b) Tooth 31 fully erupted, and periapical radiolucency was seen.

An 8-year-old girl was referred to the Department of Pediatric Dentistry, Nanjing Stomatological Hospital, with the chief complaint of intraoral swelling and pain for over a week. An examination of her medical history showed that she had suffered similar symptoms a year ago and one of the lower incisors (tooth 41) had been treated with apexification. No systematic medical condition was reported. Intraoral examination revealed mixed dentition with normal maxillary incisors and short and worn mandibular central incisors. The permanent right lateral incisor (42) had merely erupted, and only the incisal third could be seen. The primary left lower lateral incisor (72) was present, and the corresponding permanent tooth was not seen. Oral hygiene was poor. A thick layer of debris was observed on the surface of the mandibular incisors. Swelling was extensive in the lower central region. All present mandibular incisors (72, 31, 41, and 42) were mobile and sensitive to percussion/palpation. Radiographic examination revealed that the newly emerged permanent mandibular incisors (31, 41, and 42) were characterized with extremely thin layer of hard tissue, short root, and enlarged pulp space. Periapical radiolucency without caries was noticed for 31 and 42. Radiopaque material was observed in the pulp cavity of 41 (Figure 1).

Based on the symptoms, clinical and radiographic examinations, the definitive diagnosis of the patient was dentinogenesis imperfecta and apical periodontitis of the permanent lower left central incisor [16]. The objective of the short-term treatment was to relieve the pain and avoid further inflammation. Long-term treatment plan included acquiring a functional and aesthetic dentition without pain or sensitivity. Given consideration to the enlarged pulp cavity and short root of the tooth 31 with apical periodontitis, RET was recommended. Risks and benefits of RET and the traditional apexification were explained to the parents, and informed consent was obtained from the parents to perform RET.

**Figure 2:**
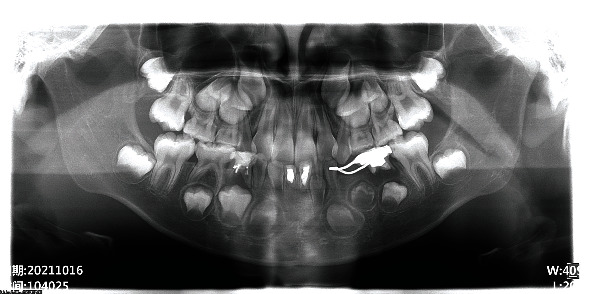
Immediate postoperative panoramic radiograph. Tooth 31 was treated with RET. Tooth 41 had been treated with apexification. Permanent mandibular incisors and canines exhibited poor formation of dental hard tissue.

At the first visit, emergent procedures were taken. Access cavity was prepared, and bloody and purulent exudate was seen. The pulp chamber was gently irrigated with 1.0% sodium hypochlorite and sterile saline. No mechanical instrumentation was performed. The affected tooth was left open for 3 days until the second visit to achieve drainage of exudate. At the second visit, intraoral swelling was significantly alleviated and the apical exudation was minimized. After isolating the tooth, pulp disinfection was performed following the American Association of Endodontists' protocol with minor modifications [17]. Briefly, the tooth was thoroughly and gently irrigated with 20 mL 1.0% sodium hypochlorite, dried, and dressed with triple antibiotic paste. Equal amount of metronidazole (Qidu Pharmaceutical Company, China), minocycline (Hanhui Pharmaceutical Company, China), and ciprofloxacin (Jingxin Pharmaceutical Company, China) was mixed and dissolved with sterile saline to make the triple antibiotic paste (TAP). Dentin bonding agent (3M ESPE, Germany) was applied on the walls of pulp chamber to avoid discoloration caused by TAP. The tooth was then temporarily restored using Caviton (GC, Japan). After four weeks, at the third visit, clinical examination revealed that the tooth was asymptomatic. Local anesthesia was performed with 2% lidocaine HCl (without epinephrine; Tiansheng Pharmaceutical Company, China). The tooth was isolated, and the temporary restoration was removed. The root canal was irrigated with sterile saline to remove the antibiotic paste. The pulp cavity was then dried with paper point. A #40 file was used to irritate the apical tissue. Very limited amount of blood was acquired through the irritation procedure, and only the apical third was filled with blood. A bioceramic material, iRoot BP (Innovative BioCeramix, Canada), was placed upon the newly formed blood clot. The tooth was then restored with SonicFill ultrasonic composite resin (Kerr, Germany). In order to examine the position of the blood clot and iRoot BP plug, while in concern that other permanent teeth might also be affected by dentinogenesis imperfecta, a panoramic radiograph was taken (Figure 2).

**Figure 3:**
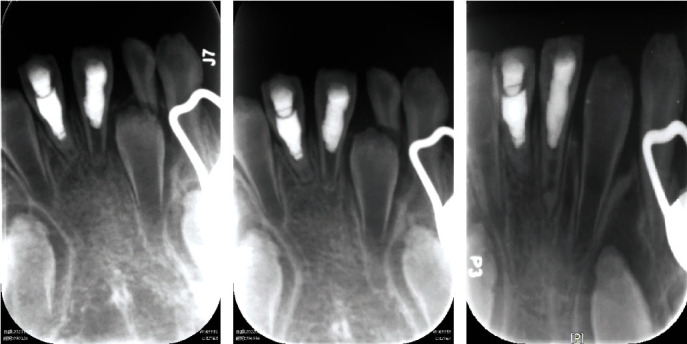
Recall radiographs of the case. (a) One-month postoperative radiograph showed alleviation of periapical radiolucency. (b) Three-month postoperative radiograph demonstrated healing of periapical lesion. Maturation of root was not evident. (c) Twelve-month postoperative recall radiograph clearly showed elongation of root and thickening of dentinal canal walls, especially when compared with tooth 41, which was treated with apexification. Dentinal bridge and closure of root apex were also obvious.

At 1-month follow-up appointment, the tooth was reported asymptomatic. X-ray examination revealed significant decrease of periapical radiolucency (Figure 3(a)). At 3-month, 7-month, and 12-month follow-up appointment, the patient reported that the tooth was asymptomatic and functional. Radiographic examination was performed at 3-month and 12-month postoperative recall, which revealed complete healing of periapical lesion, elongation of the root, closure of the root apex, and obvious dentinal bridge beneath iRoot BP plug (Figures 3(b) and 3(c)).

## 3. Discussion

Dentinogenesis imperfecta includes a series of dentin formation anomalies with different genetic alterations and phenotypic characters. Involvement of endodontic inflammation is rare in dentinogenesis imperfecta. This current clinical case reported pulpal pathosis of a tooth with dentinogenesis imperfecta due to the hypomineralized tooth structure and enlarged pulp cavity. RET was applied to control the inflammation and induce further root development. Both targets were reached successfully, showing a novel strategy for managing these challenging conditions.

A classical classification of isolated dentin diseases based on clinical phenotypes was proposed by Shields et al. [18]. Studies have shown that these different phenotypes are actually different severities of the same pathology [19–22]. All genetic mutations responsible for dentinogenesis imperfecta have been found to be located on a gene encoding DSPP. Thus, de La Dure-Molla et al. proposed a new classification to simplify diagnosis [7]. The most severe phenotype of dentinogenesis imperfecta, DGI-III, has been described with brown opalescent crown discoloration, enlarge pulp, and “shell teeth” appearance [23]. Due to the large pulp cavity and thin dentin layer, pulp exposure and pulpal inflammation could be possible [8]. This specific phenotype was first found in the triracial subpopulation of Maryland and was described as “the Brandywine isolate” [5]. Most literatures reported DGI-II phenotype in Asian populations [20, 24]. This current case reported typical “shell teeth” phenotype in an Asian patient. Early diagnosis of such cases would be beneficial as timely protection of the hard tissue with composite restorations could avoid potential pulp exposure [8].

Once pulp involvement occurs in immature permanent teeth with severe dentinogenesis imperfecta, the treatment strategies could be difficult. Most studies reported extraction for such cases [25]. Root canal treatment is impossible for such teeth with short root length and open apex. Apexification seemed a rational choice in this case. However, apexification has limited effect in inducing root development [11]. As the root remains short and the dentinal canal walls remain thin, root fracture is of high risk and extraction could be expected in the long run. In order to achieve long perseverance of the affected tooth, RET was selected for this case. RET has been proved ideal for immature teeth with pulp inflammation [26]. RET recruits stem cells of apical papilla which could survive even after pulp necrosis or apical periodontitis [13]. These stem cells together with growth factors, including DSPP, ALP, and DMP, could contribute to regeneration of pulp-dentin complex [12]. Histologic studies found cementum-like, periodontal ligament-like, and bone-like tissue in canal space after RET [10]. While whether RET induces “true” regeneration remains controversial, the increase of root length and thickening of canal walls have been observed in numerous cases [27]. In this case, obvious maturation of root was observed via radiographic examination, especially when compared with its adjacent tooth which was handled with apexification (Figure 3). With longer root and thicker canal walls, lower risks of root fracture could be expected.

In this study, TAP was used to disinfect root canal of the affected tooth. Studies have shown that TAP could remove diverse groups of facultative gram-positive and gram-negative microorganisms and provide an environment for healing of periapical lesions [28]. However, concerns about the drawbacks of TAP have also been addressed. Minocycline could cause calcium chelation from the dentin and has a demineralizing effect on dentin, leading to the brittleness of tooth [29]. Discoloration is the major drawback of TAP, which was also caused by minocycline [30]. Double antibiotic paste (DAP) could be alternatively used to prevent tooth staining [31]. Also, application of dentin bonding agent can greatly reduce the risk of tooth staining [16]. In this case, as the tooth was already seriously worn and appeared yellowish before treatment, aesthetics was not the major concern. For future cases with aesthetic requirement, DAP should be used to avoid tooth discoloration.

To our knowledge, this is the first case describing use of RET in necrotic immature permanent tooth with dentinogenesis imperfecta. Literatures have shown that DSPP plays an important role in both biological and RET-induced root maturation [32, 33]. However, expression of DSPP has been found to be impaired in teeth with dentinogenesis imperfecta [7]. Therefore, we were initially doubtful about the outcome of RET in this case. Evident root maturation illustrated by radiographic examinations indicated that relevant cells and growth factors have been successfully recruited. Findings from this study suggest that RET should be considered not only for immature permanent teeth without developmental defects but also for teeth with structure anomalies. Further studies are required to understand the mechanism of RET-induced root maturation in dentinogenesis imperfecta-affected teeth. Examination of levels and expression of growth factors associated with maturation of root as well as regeneration of pulp tissue, including DSPP, ALP, and DMP, will help reveal the biological process. Animal models should also be used to mimic the dentinogenesis imperfecta phenotype and explore the effectiveness of RET in such cases.

## 4. Conclusions

This case report shows that RET could induce root maturation in necrotic immature teeth affected by dentinogenesis imperfecta. Apart from conventional apexification, pediatric dentists and endodontists are encouraged to consider RET as a beneficial strategy for such immature permanent teeth with developmental defects of hard tissue and pulp involvements.

## Data Availability

The data used to support the findings of this work are available from the corresponding author upon request.
